# Hepatocellular Glycogen Accumulation in the Setting of Poorly Controlled Type 1 Diabetes Mellitus: Case Report and Review of the Literature

**DOI:** 10.1155/2020/9368348

**Published:** 2020-02-17

**Authors:** Atinuke Aluko, Ikponmwosa Enofe, Jacob Burch, Julie Yam, Nazia Khan

**Affiliations:** ^1^Michigan State University, Department of Internal Medicine, East Lansing, MI, USA; ^2^Sparrow hospital, Lansing, MI, USA; ^3^Michigan State University, Gastroenterology Fellowship, East Lansing, MI, USA

## Abstract

Glycogenic hepatopathy (GH) is the accumulation of glycogen in the hepatocytes and represents a rare complication in patients with diabetes mellitus (DM), most commonly type 1 DM. We present a case of a 23-year-old woman with a medical history of poorly controlled type 1 DM and gastroesophageal reflux disease (GERD) who presented with progressively worsening right-sided abdominal pain. Diagnostic workup resulted in a liver biopsy with hepatocytes that stained heavily for glycogen with no evidence of fibrosis or steatohepatitis. A diagnosis of glycogenic hepatopathy was made, and an aggressive glucose control regimen was implemented leading to resolution of symptoms and improvement in AST, ALT, and ALP. In addition to presenting this rare case, we offer a review of literature and draw important distinctions between glycogenic hepatopathy and other differential diagnoses with the aim of assisting providers in the diagnostic workup and treatment of glycogenic hepatopathy.

## 1. Introduction

Glycogenic hepatopathy, a rare occurrence usually seen in poorly controlled type 1 DM, is due to the accumulation of glycogen within the hepatocytes [1]. Patients with GH have an enlarged liver, elevated liver enzymes, and commonly present to the hospital with complaints of right upper abdominal pain [1–5]. It is crucial that this condition is diagnosed and differentiated from other conditions which present with similar symptoms, as GH is reversible unlike other differentials including nonalcoholic fatty liver disease (NAFLD) which is more commonly seen in type 2 DM [3]. Despite the increasing incidence of diabetes in the United States [6], the real incidence and prevalence of GH are unknown [1–5, 7–16], commonly being misdiagnosed or underdiagnosed. We present a case of a patient with poorly controlled type 1 diabetes who presented with abdominal pain, abnormal liver enzymes, and massive enlargement of the liver and was subsequently diagnosed with glycogenic hepatopathy.

## 2. Case Presentation

A 23-year-old woman with type 1 DM, GERD, amenorrhea (resolved) and more recently irregular menstrual periods whom presented to the emergency department with complaints of right-sided abdominal pain of 14 days duration. Pain was described as sharp in nature, intermittent, worsened by eating, and without any relieving factors. It was associated with nausea and had been progressively increasing in intensity and frequency of occurrence. She was diagnosed with type 1 DM 11 years prior, which was poorly controlled, with a mean HbA1c of 12.05% over a 2-year period. She had been admitted multiple times for DKA.

At presentation, the patient appeared well developed for her age except for a short stature and being underweight (BMI 17.54). She was tachycardic (HR 117 bpm) and tender in the right upper and lower quadrants of the abdomen without guarding or rebound tenderness. The liver was palpable 3 inches below the right costal margin. Laboratory results revealed mild DKA as evidenced by the elevated blood sugar (344 mg/dl), anion gap acidosis of 17, HCO3 of 18 mMol/L, venous pH 7.31, serum ketones < 1 : 4 dilution, and trace urine ketones. Her blood glucose reduced to 76 mg/dl after two liters of intravenous fluids. Her home insulin regimen was promptly resumed, and she did not require an insulin infusion. AST (511 mg/dl), ALT (366 mg/dl), and ALP (304 mg/dl) were elevated. Lactic acid was elevated at 8.0 mg/dL. Total bilirubin (0.4 mg/dl), albumin (4 mg/dl), INR (0.9), and platelet count (248 cells/microliter) were within normal limits. HbA1c on presentation was 11.5%. Other laboratory values including amylase and lipase were within normal limits.

**Figure 1:**
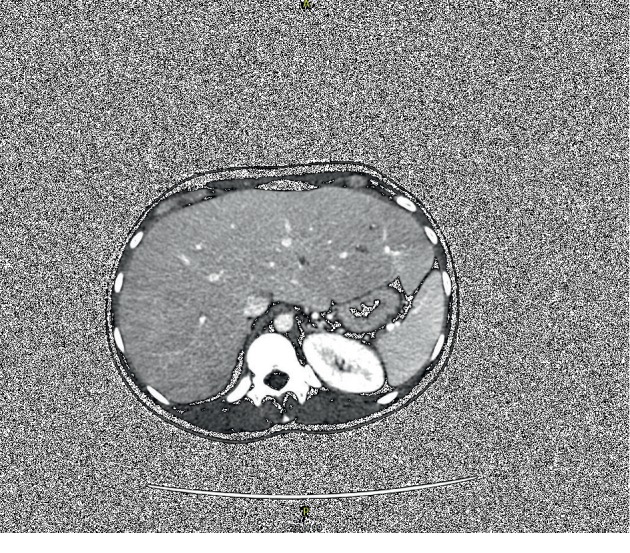
Axial commuted tomography scan, cross-sectional view showing massive hepatomegaly.

**Figure 2:**
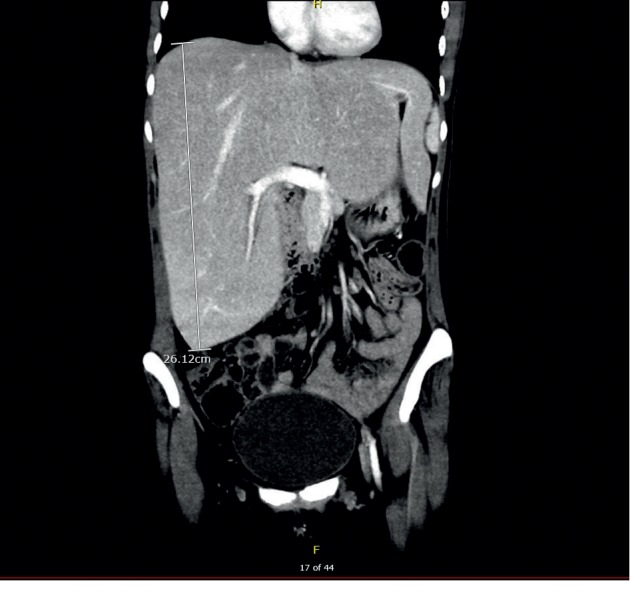
Coronal commuted tomography scan section showing an enlarged liver with maximum dimensions measuring 25.0 × 23 × 14.8 cm liver.

Commuted tomography (CT) of the abdomen and pelvis with contrast (Figures 1 and 2) showed an enlarged liver, 26 cm in full length with mass effect on adjacent structures including the inferior vena cava, stomach, duodenum, pancreas, and hepatic flexure of the colon. Abdominal ultrasound with Doppler was negative for hepatic vein thrombosis. Of note, the patient was not on any hepatotoxic medications. Laboratory workup for causes of elevated liver enzymes including: infective workup (EBV, CMV, Hepatitis A, B, and C), iron studies (normal ferritin, decreased total iron, and iron saturation), ceruloplasmin level, serum alpha-fetoprotein, and thyroid function were all within normal limits. Celiac disease had been ruled out previously in the outpatient setting with negative tissue transglutaminase antibodies and endomysial antibody. Autoimmune workup including antinuclear antibody (ANA) titer, antismooth muscle antibody, antimitochondrial antibody, and liver/kidney microsome were all negative or within normal limits. A transthoracic echocardiogram showed normal right and left ventricular functions, with a left ventricular ejection fraction of 55–60%.

**Figure 3:**
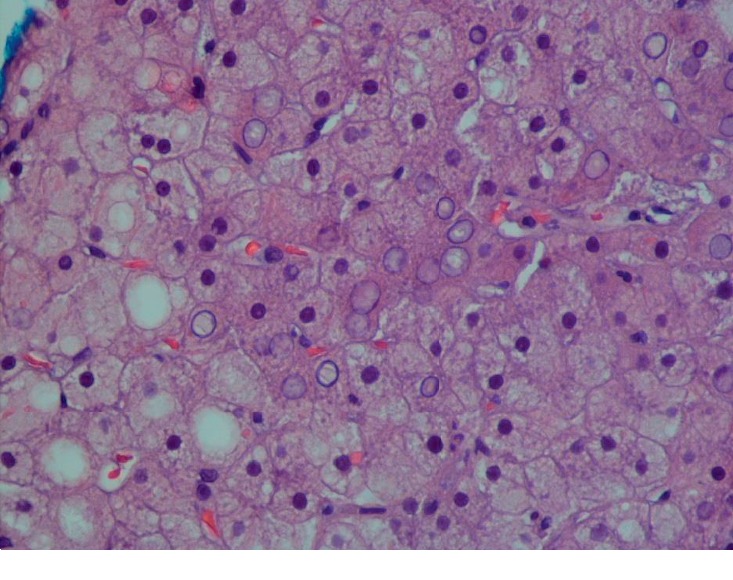
Histology of the liver showing hematoxylin and eosin (H & E) stain 40x showing pale and swollen hepatocytes.

**Figure 4:**
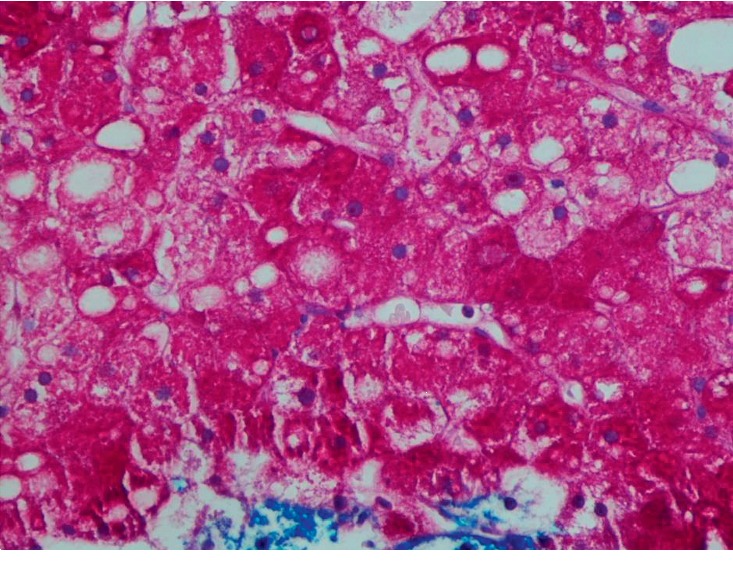
Histology of the liver with Periodic Acid Schiff (PAS) staining 40x without diastase showing hepatocytes heavily staining for glycogen.

**Figure 5:**
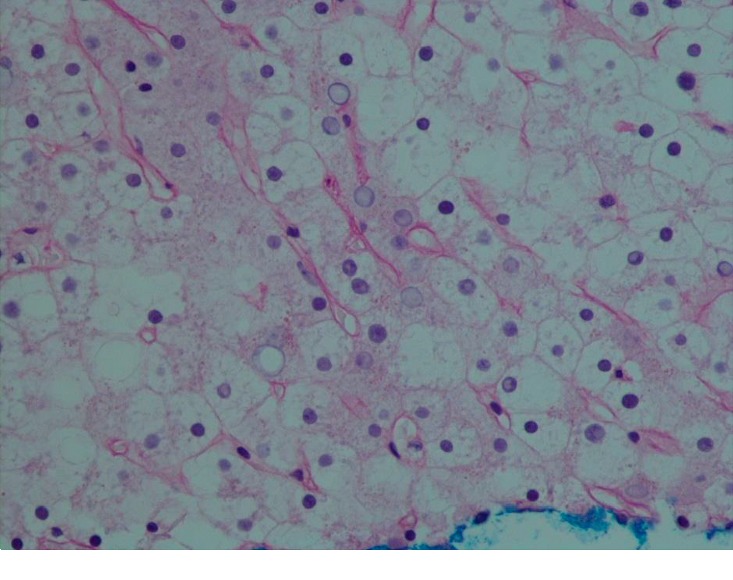
Histologic finding following periodic shift acid staining (PAS) after digestion with diastase 40x showing hepatocytes which do not stain with PAS confirming glycogen is responsible for findings.

CT-guided biopsy of the liver was done due to diagnostic uncertainty and increasing AST/ALT (Table 1). Liver biopsy showed pale and swollen hepatocytes (Figure 3) with heavy glycogen staining seen on Periodic Acid Schiff (PAS) stain (Figure 4) which was absent after digestion with diastases (Figure 5). Numerous intranuclear glycogen inclusions, as well as some patchy steatosis, were noted. There were no signs of fibrosis or increased iron deposition.

**Figure 6:**
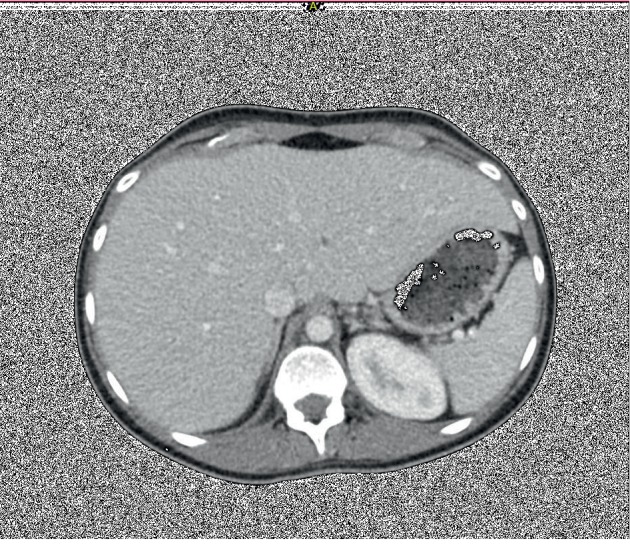
Axial commuted tomography scan, cross-sectional view showing massively enlarged liver at 12 months follow-up.

**Figure 7:**
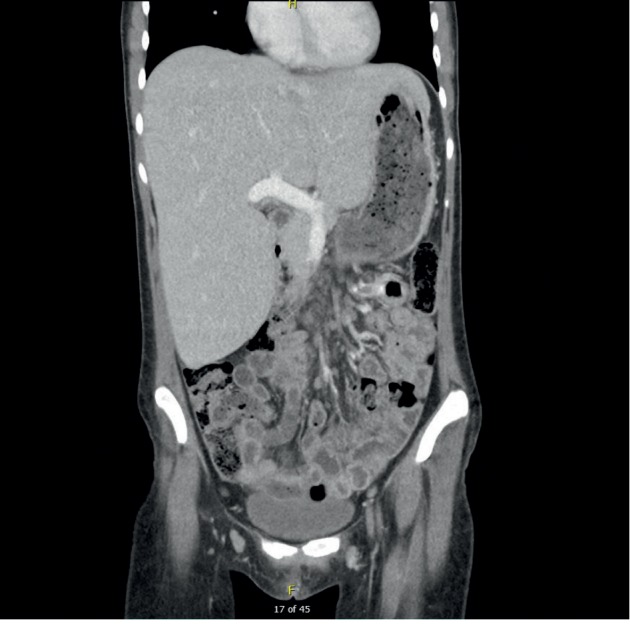
Coronal commuted tomographic scan section showing an enlarged liver with maximum dimensions measuring 25.0 × 23 × 14.8 cm at 12 months follow-up.

A diagnosis of glycogenic hepatopathy was made. She was managed symptomatically for pain and strict glycemic control was implemented. Symptoms of abdominal pain improved, and lactic acid returned to normal (1.7 mg/dl) on hospital day 3 with adequate fluid resuscitation. The patient was discharged home on hospital day 7 (Table 1) with a more aggressive insulin regimen, and follow-ups with her primary care physician (PCP) and endocrinologist were scheduled. At discharge (day 7), AST, ALT, and ALP were 237 mg/dl, 271 mg/dl, and 200 mg/dl, respectively. Unfortunately, the patient was not compliant with follow-up and was not seen for another year at which time her HbA1C was 12.6%. Liver enzymes however were within normal limits with ALT, AST, and ALP at 20 mg/dl, 35 mg/dl, and 54 mg/dl, respectively. Repeat contrasted CT abdomen twelve months later showed persistent hepatomegaly measuring 27.8 × 21.4 × 15.5 cm (Figures 6 and 7). Liver dimensions were similar to the CT findings at initial presentation. The importance of strict glycemic control was reemphasized, and she was rescheduled for follow-up with her primary care physician and endocrinologist.

## 3. Discussion

Glycogenic hepatopathy (GH) is the accumulation of glycogen in the hepatocytes [1–5, 7–11]. Mauriac first described a syndrome in the 1930s after observing children with brittle diabetes who had reduced growth, delayed puberty, Cushing-like features, hepatomegaly, and elevated liver enzymes [5]. Over time, children and adults with brittle diabetes were noted to present with some components of Mauriac syndrome, commonly hepatomegaly and elevated liver enzymes termed glycogenic hepatopathy [7]. GH is underrecognized and often confused with fatty liver disease and nonalcoholic steatohepatitis, which is more common in type 2 diabetes. The exact incidence and prevalence of the disease are unknown [1–5, 7–11]. Classically it is seen in longstanding, poorly controlled, type 1 diabetes (98% of cases) with a mean duration of 10.5 years and a mean HbA1c of 11.8 [1–3]. No gender predilection exists for developing GH [1, 4], and patients typically have a healthy body mass index [1–4].

The exact mechanism by which glycogen accumulates in the cytoplasm of the hepatocytes in patients with glycogenic hepatopathy is unknown. Higher glucose concentration activates the passive diffusion of glucose across the cell membrane by a noninsulin-dependent mechanism bypassing the insulin-dependent serum glucose transporter-2 (SGT-2) that acts to maintain glucose equilibrium between the extracellular and intracellular space [17]. This passive diffusion of glucose across the cell membrane by a noninsulin-dependent mechanism leads to increased glucose in the cytoplasm [17]. High levels of glucose in the cell results in more substrates for glycogen synthesis. Glucokinase present in the cytoplasm converts glucose to glucose-6-phosphate while glycogen synthetase, an enzyme also present in the cytoplasm of hepatocytes, converts glucose-6-phosphate to glycogen [8, 10]. A phosphatase activates glycogen synthase by dephosphorylation, and a high concentration of glucose and insulin modulate the activities of this phosphatase [18]. Thus, increased hepatic glycogenesis is thought to occur due to the presence of high cytoplasmic glucose concentration as well as the presence of exogenous insulin [3, 8–10]. The activation of phosphatase by increased cytoplasmic glucose and high levels of exogenous insulin rather than an alteration in the coding sequence of the glycogen phosphorylase gene better explain the increased activity of phosphatase seen in patients with GH [19].

Clinically, patients with GH may present in DKA with symptoms such as polyuria, polydipsia, dehydration, abdominal pain, nausea, and vomiting [18, 20]. They also have hepatomegaly (more than 90% of the time) [21], varying degree of elevated transaminases, and rarely elevated alkaline phosphatase. Elevated liver enzymes present with a hepatocellular pattern of injury rather than a cholestatic pattern [1, 2, 9], usually with AST greater than ALT [22]. This is likely due to enzyme leakage as a result of hepatocyte membrane injury [1, 19]. There is no histologic evidence to suggest cell death as a cause of elevated transaminases [22]. Hepatocellular injury in GH can present with mild elevations in transaminases to dramatically elevated values with some reported cases of liver enzymes up to 30 times the upper limit of normal [23–25]. Satyarengga et al. reported a case with peak AST of 3725 mg/dl and ALT 1049 mg/dl [24]. However, no cases of acute liver failure have been reported in the literature. As seen in our patient, elevation in lactic acid can occur in these patients [16]. The exact significance of this remains unknown [26]. However, some studies attribute this to reduced gluconeogenesis and lack of conversion of pyruvate to glucose [16]. Patients with GH do not have abnormalities in the synthetic function of the liver [2, 20]. There are a few cases of GH presenting with ascites without any signs of cirrhosis. The mechanism of ascites remains unclear [1]. Other laboratory abnormalities in patients with GH include a high amylase and/or lipase in patients with no clinical or radiographic evidence of acute pancreatitis [11]. The underlying mechanism of this is also unknown [11]. However, these findings of elevated lipase and amylase were not present in our patient.

A high index of suspicion is required in a patient with poorly controlled type 1 diabetes who presents with abdominal pain, hepatomegaly, and elevated liver enzymes [21]. There are, however, case reports of GH occurring in patients with type 2 diabetes with poor glycemic control [12, 13]; nondiabetics on high dose steroids [14]; a 3-year-old with dumping syndrome associated with gastrostomy feeding without glucose intolerance [15]; a patient who developed rapid onset hyperglycemia but had a HbA1c of 6.2% [27]; and a person with type 2 diabetes who attempted suicide with large dose of insulin glargine, treated with hypercaloric infusion [13].

The diagnosis of GH is made in patients in whom we have a high index of suspicion following a comprehensive history, physical examination, laboratory testing, and imaging studies. Liver biopsy is the gold standard for making a diagnosis of GH [21] but this is not always necessary. Liver biopsy should be performed in cases where the diagnosis is not definite, and other causes of hepatic dysfunction cannot be excluded [21]. Similarly, liver biopsy is reserved for patients who do not respond to treatment with tight glycemic control or have a persistent rise in liver enzymes despite improved glycemic control [28]. In evaluating diabetic patients with abdominal pain, hepatomegaly, and elevated liver enzymes in whom we suspect GH, history should include the duration of symptoms, how long the patient has had diabetes, and level of glycemic control. Abdominal imaging including ultrasound scan (US), CT, and gradient dual echo magnetic resonance imaging (MRI) are helpful non-noninvasive tools which, in addition to history and physical examination, can aid in the diagnosis of GH and reduce the need for invasive liver biopsies [21]. Sweetser and Kraichely reported the usefulness of CT to distinguish GH from fatty liver disease [29]. CT scan can differentiate higher liver densities in GH from lower densities seen in patients with fatty liver [29]. However, the use of CT scan is limited in cases where lower liver densities associated with fatty change occur in patients with GH [27] or cases with a smaller increase in liver density than expected, as seen in glycogen storage diseases [30, 31]. The use of CT scan in diagnosing GH may further be complicated by conditions that cause the liver to be hyperdense on imaging as seen in hemochromatosis and iodine deposition in the liver of patients who take amiodarone [28, 29]. Gradient dual echo MRI can distinguish fat deposition from edematous conditions, such as patients with acute liver injury, and from hepatitis both of which may appear as low-density areas on CT [32, 33]. The sensitivity of gradient dual echo MRI combined with other imaging modalities, like abdominal US and CT, increases in differentiating between GH and NASH thus reducing the need for a liver biopsy [27]. Unfortunately, our patient did not get a gradient dual echo MRI as this is not done at our center. Due to diagnostic uncertainty in our patient's presentation, a liver biopsy was done to confirm the diagnosis. Noninvasive tests like Fibro Sure have no use in the evaluation of GH or in differentiating GH from NASH [21]. The importance of differentiating these two clinical entities is necessary as their management and prognosis differ. All cases reported so far indicate that GH runs a benign [2, 10, 18, 19] and reversible course with tight glycemic control, while some cases of NASH progress to cirrhosis and hepatocellular carcinoma [34].

Histologically, accumulation of glycogen in the hepatocytes is pathognomonic. Hepatocytes are pale and swollen. There is absent to minimal inflammation and no fatty changes or spotty lobular necrosis. The architecture of the liver is preserved without any significant fibrosis. Glycogen accumulation demonstrated using PAS-diastase staining shows abundant cytoplasmic glycogen deposits which disappear after digestion with diastase [1, 20].

Liver enzymes usually normalize within 2–14 weeks of tight glycemic control, mostly achieved by subcutaneous insulin administration [10, 35, 36]. In two case reports, patients with poorly controlled type 1 diabetes and frequent episodes of hypoglycemia diagnosed with GH had a resolution of GH following pancreatic transplantation for other indications [19]. GH can recur if there is a relapse in poor glycemic control [9]. The level of glycemic control required for normalization of transaminitis and complete resolution of hepatomegaly remains unclear. However, few cases have reported decrease or normalization of liver enzymes and improvement of symptoms with a modest drop in HbA1c, without the need for aggressive insulin treatment [22, 29]. Cha et al. reported the resolution of transaminitis in patients with HbA1c above 11%. Similarly, Parmar et al. reported relief of abdominal pain and a decline in liver enzymes in their patient with a decrease in HbA1c of 0.6% (13.7% to 12.7%) [25].

In our patient, despite suboptimal glucose control, transaminitis resolved and liver enzymes were within normal limits a year later, which points towards the benign course of the disease. Follow-up repeat CT of the abdomen did not show a reduction in liver size which could be explained by the suboptimal glucose control, noncompliance with insulin regimen evident by HbA1c of 12.6%, and temporary loss to follow-up. The patient remains at risk for relapse due to inadequate glycemic control [9, 37].

## 4. Conclusion

Patients with GH usually present with nonspecific symptoms that may lead to a delay in diagnosis. In patients with type 1 DM with elevated liver enzymes with or without hepatomegaly, glycogenic hepatopathy should be considered as a differential diagnosis. A high index of suspicion by clinicians can lead to prompt diagnosis and potentially decrease the need for unnecessary testing in this patient population. It is important to distinguish GH from NAFLD for prognostication and management with the former following a benign cause and the latter progressing to cirrhosis in some cases. We advocate more research to understand better risk as well as potentiating and/or protective factors associated with the development of GH.

## Figures and Tables

**Table 1:** Trends in liver enzyme during hospitalization and at follow-up a year later.

Trends in liver enzymes											
Days	0	1	2	3	4	5	6	7	14	24	365
AST	511	371	307	275	617	764	630	367	237	101	23
ALT	366	284	249	236	321	374	355	320	271	91	33
